# Phase Field Study on the Spinodal Decomposition of β Phase in Zr–Nb-Ti Alloys

**DOI:** 10.3390/ma16082969

**Published:** 2023-04-08

**Authors:** Kun Yang, Yanghe Wang, Jingjing Tang, Zixuan Wang, Dechuang Zhang, Yilong Dai, Jianguo Lin

**Affiliations:** 1 School of Materials Science and Engineering, Xiangtan University, Xiangtan 411105, China; 2 Department of Materials Science and Engineering, Southern University of Science and Technology, Shenzhen 518055, China; 3 Key Laboratory of Materials Design and Preparation Technology of Hunan Province, Xiangtan University, Xiangtan 411105, China

**Keywords:** Zr-Nb-Ti alloy, spinodal decomposition, microstructure evolution, phase field method

## Abstract

In this study, a phase field method based on the Cahn–Hilliard equation was used to simulate the spinodal decomposition in Zr-Nb-Ti alloys, and the effects of Ti concentration and aging temperature (800–925 K) on the spinodal structure of the alloys for 1000 min were investigated. It was found that the spinodal decomposition occurred in the Zr-40Nb-20Ti, Zr-40Nb-25Ti and Zr-33Nb-29Ti alloys aged at 900 K with the formation of the Ti-rich phases and Ti-poor phases. The spinodal phases in the Zr-40Nb-20Ti, Zr-40Nb-25Ti and Zr-33Nb-29Ti alloys aged at 900 K were in an interconnected non-oriented maze-like shape, a discrete droplet-like shape and a clustering sheet-like shape in the early aging period, respectively. With the increase in Ti concentration of the Zr-Nb-Ti alloys, the wavelength of the concentration modulation increased but amplitude decreased. The aging temperature had an important influence on the spinodal decomposition of the Zr-Nb-Ti alloy system. For the Zr-40Nb-25Ti alloy, with the increase in the aging temperature, the shape of the rich Zr phase changed from an interconnected non-oriented maze-like shape to a discrete droplet-like shape, and the wavelength of the concentration modulate increased quickly to a stable value, but the amplitude decreased in the alloy. As the aging temperature increased to 925 K, the spinodal decomposition did not occur in the Zr-40Nb-25Ti alloy.

## 1. Introduction

Titanium and its alloys have a low density, high specific strength, low elastic modulus and good corrosion resistance, and as such they have been widely used in aerospace, biomedicine and other fields. As biological implanting materials, biocompatibility and biomechanical compatibility are also very important properties. Traditional α titanium alloys with an HCP structure, such as the Ti-6Al-4V alloy, have excellent comprehensive mechanical properties; however, they comprise Al and V elements, which have been proven to be biotoxic after being introduced into the human body, meaning that they result in a range of health problems [1]. In terms of biomechanical compatibility, in comparison to other implantable metal materials, conventional titanium alloys yield a relatively low elastic modulus, and this is much higher than that of the human bones. Thus, after being implanted into the human body as a hard implant, they may cause a “stress-shielding effect”.

Generally, Ti alloys are classified into three types of alloys according to their phase constituents and microstructures, i.e., a-type Ti alloys, (a+β)-type Ti alloys and β-type Ti alloys. It has been well documented that the additions of some biocompatible alloy elements, such as Nb [2], Mo [3], Ta [4], Zr [5], Cu [6], Fe [7] and Sn [8,9], etc., can effectively promote the β phase stability of Ti alloys, and metastable β titanium alloys with a BCC structure can be obtained at room temperature [10]. Compared to the conventional titanium alloys, β titanium alloys not only have good biocompatibility, but also have an extremely low elastic modulus and high strength. Therefore, a variety of β titanium alloys have been developed in recent years, such as Ti-Nb-based alloys [11,12,13,14], Ti-Mo-based alloys [3,15], Ti-Zr-based alloys [16] and Ti-Ta-based [17,18] alloys, etc.

For a β titanium alloy, the strengthening methods mainly include solid solution, precipitation or fine grain strengthening [19,20,21]. However, these methods usually lead to an increase in the elastic modulus of the alloys while increasing the strength of the alloys. A spinodal decomposition refers to a homogeneous phase transition, where a supersaturated solid solution decomposes into two phases which possess the same structure but differ in composition on account of the instability of the solid solution [13,22]. Spinodal decomposition rapidly forms two phases with the same crystal structure by uphill diffusion; this does not require nucleation, which can improve the strength of the alloys dramatically without increasing their elastic modulus [23,24].

Clearly, it can be seen from the phase diagram that there is a large miscibility gap in the solid solution region for the Zr-Nb-Ti alloy system [25], so the spinodal decomposition may occur at a specific temperature for the Zr-Nb-Ti solid solution of a particular composition. Accordingly, a Zr-Nb-Ti alloy strengthened by a spinodal structure with a high strength and low elastic modulus can be obtained.

Previous studies show that spinodal strengthening effects are in close connection with the amplitude, wavelength, volume fraction and shape of the decomposition products [26,27]. Many documents confirm that the amplitude, wavelength, volume fraction and shape of the decomposition products are usually affected by the composition of the alloys and the aging temperature in the alloys. Therefore, it is helpful to study the growth kinetics process of spinodal phases in order to regulate the strengthening effects of spinodal decomposition in the alloy. However, few studies have been conducted on the responses of Zr-Nb-Ti alloys to aging treatments at different temperatures.

It is useful to use phase field simulation to predict the evolution of the microstructure [28]. Moreover, the simulation results can visually show the evolution of the microstructure during the spinodal decomposition process at any time and reveal the effects of the composition of the alloy, aging temperature, aging time and external field on the spinodal structure. However, no study has yet used the phase field method to simulate the spinodal decomposition process of Zr-Nb-Ti alloys. Thus, it is meaningful to study the spinodal microstructure evolution and structural characteristics of Zr-Nb-Ti alloys using the phase field method, which can provide theoretical guidance to design novel implantable β type Zr-Nb-Ti biomedical alloys strengthened by spinodal decomposition.

In view of this, in the current work, the spinodal decomposition in Zr-Nb-Ti alloys was simulated by applying the phase field method based on the Cahn–Hilliard equation, and the nonlinear Cahn–Hilliard partial difference equation was solved by using the finite element method. Accordingly, we applied the phase field method to simulate the morphology, wavelength, amplitude and volume fraction of the spinodal phases. Eventually, the effects of the Ti content and aging temperature on the morphology and volume fraction of the spinodal phases and the wavelength and amplitude of the concentration modulate were discussed. The aim of the study was to figure out the kinetics characteristics of the spinodal decomposition in Zr-Nb-Ti alloys, making it easy to regulate the microstructure of spinodal phases to obtain alloys with improved comprehensive mechanical properties.

## 2. Phase Field Model

### 2.1. Cahn–Hilliard Equation

The Cahn–Hilliard diffusion equation can be used to study the spinodal decomposition in Zr-Nb-Ti alloys, which are described as follows:

(1) $$\frac{1}{V_{m}}\frac{\partial c_{Zr}}{\partial t}=\nabla \cdot \left(M_{Nb}\nabla \frac{\delta G}{\delta c_{Zr}}\right)$$

(2) $$\frac{1}{V_{m}}\frac{\partial c_{Nb}}{\partial t}=\nabla \cdot \left(M_{Zr}\nabla \frac{\delta G}{\delta c_{Nb}}\right)$$

where $c_{Zr}$ and $c_{Nb}$ are the mole fractions of Zr and Nb atoms, respectively; $M_{Nb}$ and $M_{Zr}$ are the migration rates of the Zr and Nb atoms, respectively; *V_m_* is the mole volume; and G is the Gibbs free energy of the alloys.

The migration rate of the Zr and Nb atoms, $M_{Nb}$ and $M_{Zr}$, can be written as follows:

(3) $$M_{Nb}=\frac{1}{RT}\exp\left(\frac{Q_{Nb}}{RT}\right)$$

(4) $$M_{Zr}=\frac{1}{RT}\exp\left(\frac{Q_{Zr}}{RT}\right)$$

where $Q_{Nb}$ and $Q_{Zr}$ are the activation energies of the Nb and Zr atoms, respectively, which are expressed as follows:

(5) $$Q_{Nb}=\left(1-c_{Zr}-c_{Nb}\right)Q_{Ti,Nb}+\left(1-c_{Zr}-c_{Nb}\right)\left(c_{Zr}Q_{Ti,Zr,Nb}+c_{Nb}Q_{Ti,Nb,Nb}\right)+c_{Zr}Q_{Zr,Nb}+c_{Zr}c_{Nb}Q_{Zr,Nb,Nb}+c_{Nb}Q_{Nb,Nb}$$

(6) $$Q_{Zr}=\left(1-c_{Zr}-c_{Nb}\right)Q_{Ti,Zr}+\left(1-c_{Zr}-c_{Nb}\right)\left(c_{Zr}Q_{Ti,Zr,Zr}+c_{Nb}Q_{Ti,Nb,Zr}\right)+c_{Zr}Q_{Zr,Zr}+c_{Zr}c_{Nb}Q_{Zr,Nb,Zr}+c_{Nb}Q_{Nb,Zr}$$

where $Q_{Ti,Nb}$, $Q_{Ti,Zr}$, $Q_{Zr,Nb}$, $Q_{Zr,Zr}$, $Q_{Nb,Zr}$, $Q_{Ti,Zr,Nb}$, $Q_{Ti,Nb,Nb}$, $Q_{Zr,Nb,Nb}$, $Q_{Ti,Zr,Zr}$, $Q_{Ti,Nb,Zr}$ and $Q_{Zr,Nb,Zr}$ are thermodynamic parameters, which generally appear linear to the temperature.

The total Gibbs energy of the alloys can be described as follows:

(7) $$G=F^{v}+F_{i}^{\nabla }+F^{el}=\int_{V}{(f}^{v}+f_{i}^{\nabla }+f^{el})\;dV(i=Zr,Nb)$$

where $F^{v}$, $F_{i}^{\nabla }$ and $F^{el}$ refer to the volume free energy, the interface energy and the elastic strain energy, respectively, and $f^{v}$, $f_{i}^{\nabla }$ and $f^{el}$ refer to the densities of the volume free energy, the interface energy and the elastic strain energy, respectively.

For the Zr-Nb-Ti alloy, the density of the volume free energy can be expressed as follows:

(8) $$f^{v}=G_{Zr}^{\phi }c_{Zr}+G_{Nb}^{\phi }c_{Nb}+G_{Ti}^{\phi }c_{Ti}+RT\left(c_{Zr}lnc_{Zr}+c_{Nb}lnc_{Nb}+c_{Ti}\ln c_{Ti}\right)+G^{\phi }$$

where $G^{\phi }$ is the excess Gibbs energy of the element, which can be expressed as follows:

(9) $$G^{\phi }=c_{Zr}c_{Nb}L_{Zr,Nb}+{c_{Zr}c}_{Ti}L_{Ti,Zr}+c_{Nb}c_{Ti}L_{Ti,Nb}+c_{Zr}c_{Nb}c_{Ti}\left(c_{Ti}L_{Ti,Zr,Nb}^{0}+c_{Nb}L_{Ti,Zr,Nb}^{1}+c_{Zr}L_{Ti,Zr,Nb}^{2}\right)$$

where $c_{Ti}=1-c_{Zr}-c_{Nb}$ is the mole fraction of the Ti atom; $L_{Zr,Nb}$, $L_{Ti,Zr}$ and $L_{Ti,Nb}$ refer to the interaction coefficients between Zr and Nb, Ti and Zr, and Ti and Nb, respectively; and $L_{Ti,Zr,Nb}^{0}$, $L_{Ti,Zr,Nb}^{1}$ and $L_{Ti,Zr,Nb}^{2}$ refer to the interaction coefficients of Zr, Nb and Ti.

In the present work, we use the gradient energy density in the phase field model of the gradient energy contribution to the total free energy in the following form [28,29]:

(10) $$f_{Zr}^{\nabla }=K_{Zr}{\left({\nabla c}_{Zr}\right)}^{2}$$

(11) $$f_{Nb}^{\nabla }=K_{Nb}{\left({\nabla c}_{Nb}\right)}^{2}$$

where $K_{Zr}$ and $K_{Nb}$ refer to the gradient energy coefficients of Zr and Nb atoms, respectively, whose expressions are as follows:

(12) $$K_{Zr}=\frac{1}{2}a^{2}L_{Ti,Zr}$$

(13) $$K_{Nb}=\frac{1}{2}a^{2}L_{Ti,Nb}$$

Regarding the elastic strain energy, because the two phases obtained in spinodal decomposition have exactly the same crystal structure with a coherent interface between them, the elastic strain energy contribution is ignored in the calculation for simplicity. As a result, the final Cahn–Hilliard equation can be obtained as follows:

(14) $$\frac{\partial c_{Zr}}{\partial t}=\nabla \cdot M_{Zr}\nabla \left(\frac{\partial f_{0}}{\partial c_{1}}-2K_{Zr}\nabla^{2}c_{Zr}\right)$$

(15) $$\frac{\partial c_{Nb}}{\partial t}=\nabla \cdot M_{Nb}\nabla \left(\frac{\partial f_{0}}{\partial c_{2}}-2K_{Nb}\nabla^{2}c_{Nb}\right)$$

### 2.2. Simulation Conditions

We performed two-dimensional (2D) simulations using 64 × 64 computational grids, and the dimension of the model was 60 × 60 nm^2^. We assumed periodic boundary conditions. The Cahn–Hilliard equations were solved using the conventional finite-difference method with commercial Comsol Multiphysics software.

The boundary conditions of the concentration field $c_{i}(i=1,\;2)$ are as follows:

(16) $$n\cdot \nabla c_{i}=0$$

The initial conditions for the concentration field $c_{i}(i=1,\;2)$ are as follows:

(17) $$c_{i}=c_{i0}(i=1,\;2)$$

The parameters [25,30,31] used in the simulation of the spinodal decomposition in the Zr-Nb-Ti alloys are gathered in Table 1.

## 3. Results and Discussion

### 3.1. Effects of the Concentration of Ti on the Spinodal Decomposition of Zr-Nb-Ti Alloys

To study the effects of Ti concentration on the spinodal decomposition of the Zr-Nb-Ti alloys, we simulated the spinodal decomposition process of the alloys with different Ti concentrations (Zr-33Nb-29Ti, Zr-40Nb-25Ti and Zr-40Nb-20Ti) by using the phase field method at a temperature of 900 K, and the effects of Ti concentration on the morphology, amplitude and wavelength of the spinodal structure are discussed.

**Figure 1:**
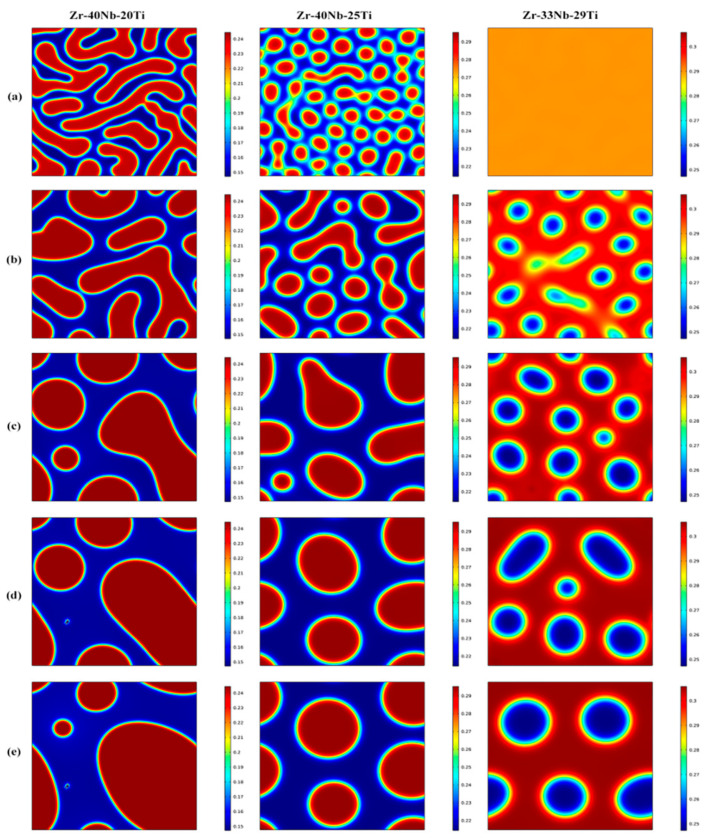
The microstructure evolution of the Zr-33Nb-29Ti, Zr-40Nb-25Ti and Zr-40Nb-20Ti alloys aged at 900 K for different times (**a**) 20 min, (**b**) 100min, (**c**) 500 min, (**d**) 1000 min and (**e**) 2000 min (the blue region refers the Ti-rich phase and the red region refers the Ti-poor phase).

Figure 1 shows the morphology evolution of the Zr-Nb-Ti alloys with different Ti concentrations annealed at 900 K for 20 to 2000 min. It is clear that the spinodal decomposition occurred in the three alloys with the formation of the modulated structure containing Ti-rich phases and Ti-poor phases. Ti concentration had an important influence on the morphology of the microstructure of the alloys. For the Zr-40Nb-20Ti alloy, the Ti-rich phase presented an interconnected non-oriented maze-like shape in the early aging period. With the extension of the aging time, the amount of the Ti-rich phase decreased but its size increased. Moreover, the shape of the Ti-rich phases gradually changed from interconnected maze-like shapes to discrete elliptical or round shapes to reduce the interface energy due to the increase in the size of the phase.

For the Zr-40Nb-25Ti alloy, the Ti-rich phases were in a discrete droplet-like shape in the early aging stage. With the increase in aging time, the discrete fine droplet-like phases were constantly fused together, resulting in an increasing size and a decreasing number of the Ti-rich phases, but the Ti-rich phases always maintained the discrete droplet-like shape.

In contrast to the first two alloys, the occurrence of spinodal decomposition in the Zr-33Nb-29Ti alloy needed a longer time at 900 K. After being aged at 900 K for 100 min, the spinodal phases can be obviously observed in the Zr-33Nb-29Ti alloy. The Ti-poor phases were in the discrete droplet-like shape, embedding in the substrate of the Ti-rich phase of the Zr-33Nb-29Ti alloy, forming a distinct droplet star spot pattern. With the increase in aging time, the amount of the droplet star-like phases decreased, but their size increased and their shape was still elliptical or round.

To quantify the effect of Ti content on the amplitude and wavelength of the concentration modulate produced by spinodal decomposition in the Zr-Nb-Ti alloy, we calculated the molar fraction of Ti atoms at different positions along the horizontal direction in the Zr-Nb-Ti alloy aged at different times using the phase field method.

**Figure 2:**
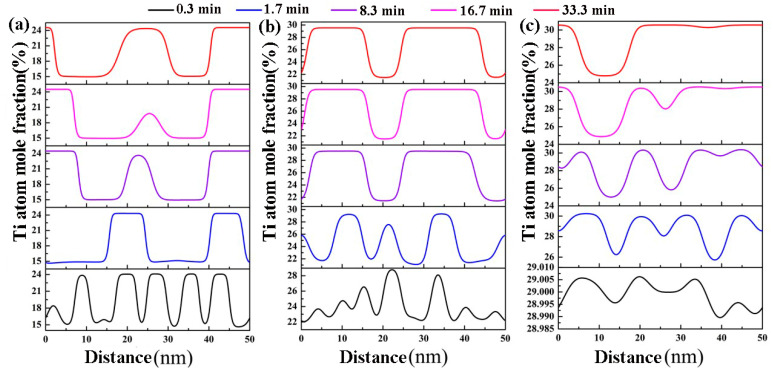
The Ti concentration fluctuation curve along the horizontal direction of the Zr-Nb-Ti alloys aged at 900 K for different times: (**a**) Zr-33Nb-29Ti, (**b**) Zr-40Nb-25Ti and (**c**) Zr-40Nb-20Ti.

Figure 2 represents the Ti concentration modulate curve along the horizontal direction in the Zr-40Nb-20Ti, Zr-40Nb-25Ti and Zr-33Nb-29Ti alloys aged at 900 K for 0.3, 1.7, 8.3, 16.7 and 33.3 min, respectively. It is obvious that the change in the molar fraction of Ti atoms is divided into three stages as follows:

The first stage: the Ti concentration fluctuates in a small length range, corresponding to the stage of the rapid formation of the Ti-rich spinodal phases.

The second stage: The amplitude of the Ti concentration fluctuation reaches the equilibrium value quickly, but its wavelength is basically not widened, corresponding to the stage of the rapid growth of the Ti-rich phases. In this stage, the number of the Ti-rich phases decreases, but the size of the phases quickly increases to an equilibrium value, resulting in the increase in the interface energy of the Zi-Nb-Ti system.

The third stage: The amplitude of the Ti concentration fluctuations basically maintains a stable value, but the wavelength is constantly broadening, corresponding to the coarsening of the Ti-rich phases. In this stage, the shape of the Ti-rich phases tends to be elliptic or circular to reduce the interface energy of the alloy system.

It can also be found that Ti concentration had an important influence on the amplitude and wavelength of the spinodal structures of the alloys. With the increase in Ti concentration, the wavelength increased, but the amplitude decreased in the three alloys. At the aging temperature of 900 K, the equilibrium concentrations of the spinodal phases of the Zr-40Nb-20Ti, Zr-40Nb-25Ti and Zr-33Nb-29Ti alloys were $c_{rich-Ti}=0.2455$ and $c_{poor-Ti}=0.1497,c_{rich-Ti}=0.2954$ and $c_{poor-Ti}=0.2148$, and $c_{rich-Ti}=0.3056$ and $c_{poor-Ti}=0.2479$, respectively.

### 3.2. Effects of Aging Temperature and Time on the Spinodal Decomposition of Zr-Nb-Ti Alloys

Limited to the length of the article, we only chose one alloy with titanium content in the middle value, and the results indicated that temperature also plays an important role in the evolution of the spinodal structure of the Zr-40Nb-25Ti alloy, and the other two alloys should also have similar results. According to the cross section of the Zr-Nb-Ti phase diagram at 923 K, there is a large miscibility gap in the β solid solution region. Thus, we selected aging temperatures ranging from 800 K to 925 K, and simulated the spinodal structure evolution of the Zr-40Nb-25Ti alloy aged at 800, 825, 850, 875, 900 and 925 K. The effects of the aging time and temperature on the spinodal decomposition of the Zr-40Nb-25Ti alloy are discussed.

**Figure 3:**
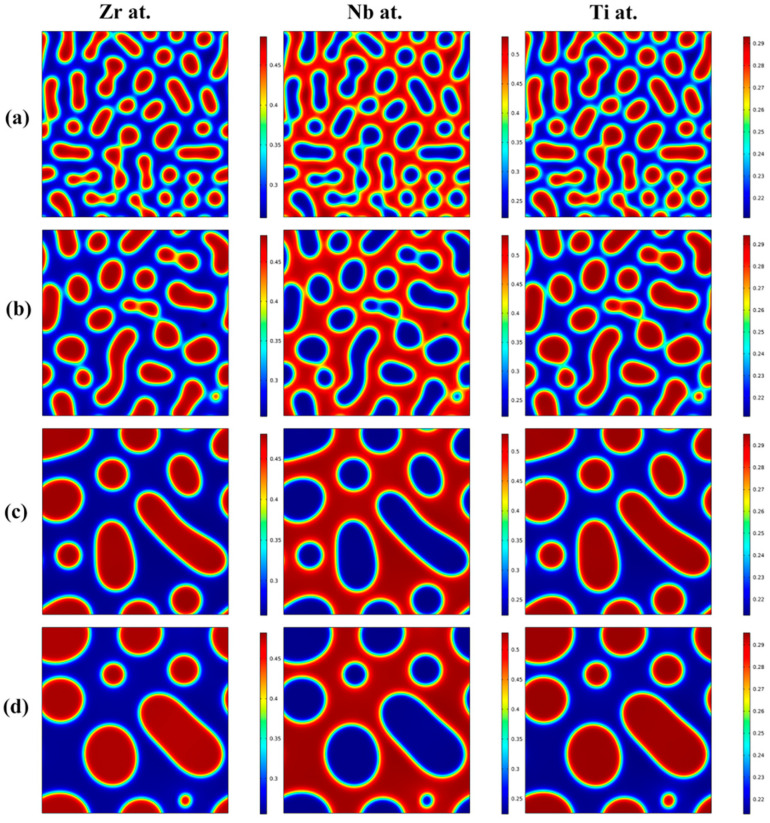
The morphology evolution of the Zr-rich, Nb-rich and Ti-rich phases with aging time increasing for the Zr-40Nb-25Ti alloy aged at 900 K for different times: (**a**) 50 min, (**b**) 100 min, (**c**) 500 min and (**d**) 1000 min.

Figure 3 shows the morphology evolution of the Zr-rich, Nb-rich and Ti-rich phases with increasing aging time for the Zr-40Nb-25Ti alloy aged at 900 K for different times. It is clear that the morphology evolution of the Zr-rich phase was similar to that of the Ti-rich phase with the increase in aging time. The two phases were both in discrete droplet-like shapes, embedding in the Nb-rich substrate. With the increase in aging time, the discrete spinodal phases continued to fuse together, leading to an increase in their size but decrease in their number, and the spinodal phases always maintained elliptical or round shapes.

**Figure 4:**
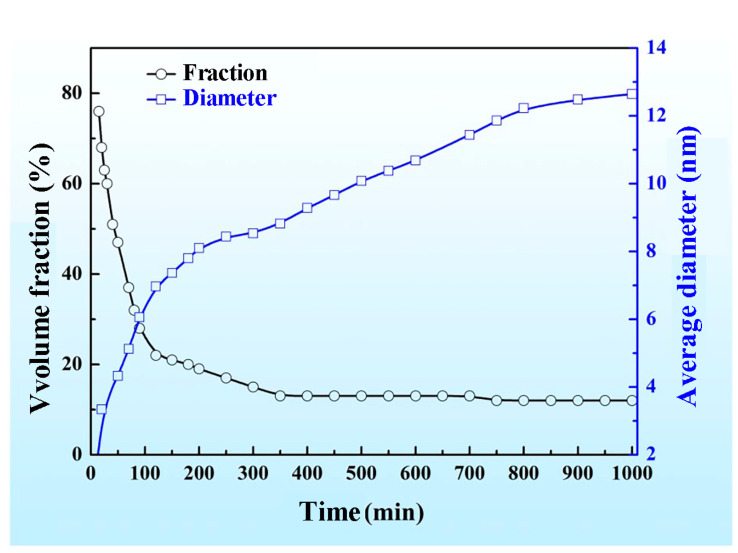
The change in the number and volume fraction of Zr-rich phases and Ti-rich phases in the Zr-40Nb-25Ti alloy aged at 900 K as a function of aging time.

To more intuitively indicate the change in the size and the volume fraction of the spinodal phases with the aging time increasing, we calculated the diameter and volume fraction of the droplet-like Zr-rich and Ti-rich phases, and the change in the mean diameter and the volume fraction of the two phases as a function of the aging time is shown in Figure 4. It is clear that the amount of the droplet-like phases dropped rapidly in the initial aging period; then, the decline rate slowed down and eventually tended to a stable value. In contrast, the size of the droplet-like phases rapidly increased in the initial aging period, and then the increase rate slowed down.

**Figure 5:**
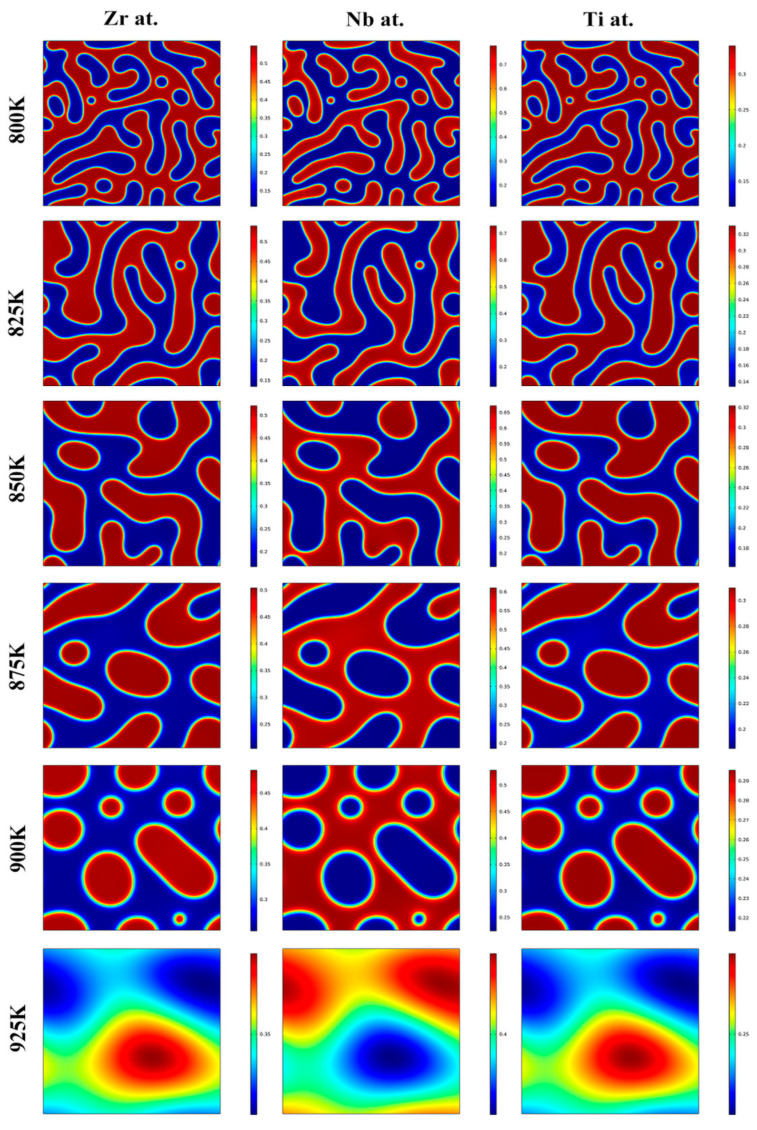
The morphology evolution of the Zr-40Nb-25Ti alloy aged at temperatures in the range of 800 K to 925 K for 1000 min.

Moreover, the morphology evolution of the Zr-40Nb-25Ti alloy aged at 800, 825, 850, 875, 900 and 925 K was simulated, and the effects of the aging temperature on it is discussed. Figure 5 shows the morphology evolution of the Zr-40Nb-25Ti alloy aged at the different temperatures. It is clear that the Ti-rich phases (marked in red) and Ti-poor phases (marked in blue) exhibited a similar morphology in the Zr-40Nb-25Ti alloy. With the aging temperature increasing from 800 K to 900 K, the rate of the spinodal decomposition of the Zr-40Nb-25Ti alloy increased. As the aging temperature further increased to 925 K, no spinodal decomposition occurred in the Zr-40Nb-25Ti alloy. The size of the spinodal phases increased with the aging temperature, reaching a maximum value of about 10 nm at 875 K, and with the aging temperature further increasing, the size of the spinodal phases almost no longer increased. Furthermore, the aging temperature also had an important effect on the morphology of the spinodal phases. With the aging temperature increasing, the shape of the spinodal phases changed from the initial interconnected unoriented maze-like shape to the discrete droplet-like shape, and the amount of the spinodal phases constantly decreased.

**Figure 6:**
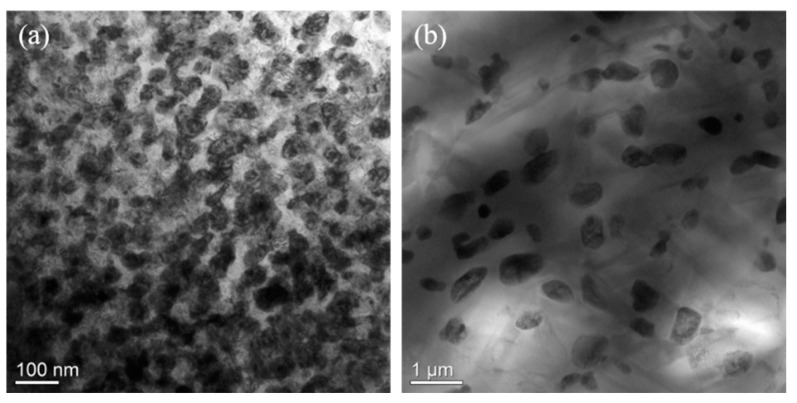
TEM bright images of the Zr-40Nb-25Ti alloy samples (**a**) annealed at 850 K and (**b**) annealed at 900 K.

To verify the simulations, we prepared a Ti alloy with the nominal composition of Zr-40Nb-25Ti by melting pure Zr (99.9%), pure Nb (99.9%) and pure Ti (99.9%) in a vacuum arc melting furnace. The alloy samples were first homogenized at 1373K for 0.5h, and then annealed at 850K and 900K, respectively. The microstructures of the samples annealed at the two temperatures were characterized by using transmission electron microscopy (TEM, JEM-2100, JEOL Ltd., Tokyo, Japan). Figure 6 illustrates the TEM bright images of the alloy samples annealed at 850 K and 900 K. It is clear that alternating dark and bright regions existing in the annealed samples, indicating the occurrence of the spinodal decomposition in the two samples. The morphology of the spinodal phases in the sample annealed exhibited an interconnected maze-like shape, while the morphology of the spinodal phases changed to the discrete droplet-like shape as the annealing increased to 900 K. The result is in good agreement with the simulation.

**Figure 7:**
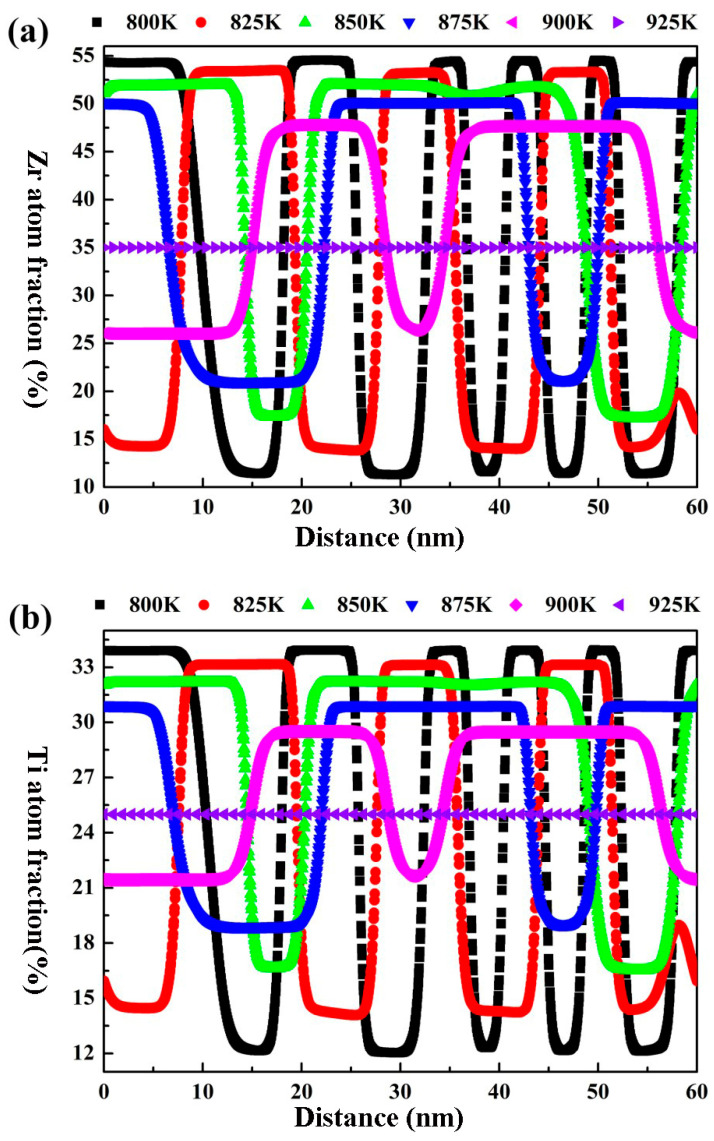
The Ti and Zr concentration fluctuation curves along the horizontal direction of the Zr-40Nb-25Ti alloy aged at different temperatures for 1000 min. (**a**) Zr concentration and (**b**) Ti concentration.

In order to clearly indicate the effect of the aging temperature on the amplitude and wavelength of the spinodal structure of the Zr-40Nb-25Ti alloy, the molar fraction of Zr and Ti atoms at different positions along the horizontal direction in the Zr-40Nb-25Ti alloy aged at different temperatures for 1000 min were calculated, and the results are shown in Figure 7. It can be seen that the changes in the amplitude and wavelength on the concentration fluctuation curves for Zr and Ti atoms exhibited similar trends with the aging temperature increasing. The amplitude on the Ti concentration fluctuation curve decreased with the aging temperature increasing. As the aging temperature increased to 925 K, the amplitude was equal to 0, implying that no spinodal decomposition occurred in the Zr-40Nb-25Ti alloy aged at that temperature. Meanwhile, the wavelength of the Ti concentration fluctuation curve increased with the aging temperature increasing, reaching its maximum value in the alloy aged at 875K, and then tended to a stable value as the aging temperature further increased. As a result, the temperature interval for the spinodal decomposition in the Zr-40Nb-25Ti alloy was in the range of 800 K to 900 K, and the morphology, amplitude and wavelength of the spinodal structure can be adjusted by changing the aging temperature.

## 4. Conclusions

In the present work, the spinodal decomposition of the β phase in the Zr-Nb-Ti alloy system aged at 800–925 K was simulated using the phase field method, and the effects of Ti concentration and aging temperature on the spinodal structure of the alloys were investigated. The main conclusions are drawn as follows:

(1) The spinodal decomposition occurred in the Zr-40Nb-20Ti, Zr-40Nb-25Ti and Zr-33Nb-29Ti alloys aged at 900 K with the formation of the Ti-rich phases and Ti-poor phases.
(2) The Ti concentration has important effects on the morphology of the spinodal phases and the wavelength and the amplitude of the concentration modulation. The spinodal phases in the Zr-40Nb-20Ti, Zr-40Nb-25Ti and Zr-33Nb-29Ti alloys aged at 900 K were an interconnected non-oriented maze-like shape, a discrete droplet-like shape and a clustering sheet-like shape in the early aging period, respectively. Increasing the Ti concentration of the alloy increased the wavelength of the concentration modulation; decreasing the Ti concentration decreased the amplitude.
(3) The aging temperature also has important effects on the morphology of the spinodal phases and on the wavelength and amplitude of the concentration modulation. For the Zr-40Nb-25Ti alloy, with the increase in the aging temperature, the shape of the Zr-rich phase changed from an interconnected non-oriented maze-like shape to a discrete droplet-like shape, and the wavelength of the concentration modulate increased quickly to a stable value, but the amplitude decreased in the alloy.

## Figures and Tables

**Table 1:** The parameters used in simulation of the spinodal decomposition in Zr-Nb-Ti alloys [25,30,31].

Parameters	Values
T (K)	850 875 900
$G_{Ti}^{\phi }$ (J/mol)	−1272.064 + 134.71418 T − 25.5768 TlnT − 0.663845 $\times \;10^{-3}\;T^{2}$ − 0.27880 $\times \;10^{-6}\;T^{3}$ + 7208 $T^{-1}$
$G_{Nb}^{\phi }$ (J/mol)	−8519.353 + 142.045475 T − 26.4711 TlnT + 2.03475 $\times \;10^{-4}\;T^{2}$ − 3.5012 $\times \;10^{-7}\;T^{3}$ − 93,399 $T^{-1}$
$G_{Zr}^{\phi }$ (J/mol)	−525.539 + 124.9457 T − 25.607406 TlnT − 3.40084 $\times \;10^{-4}\;T^{2}$ − 9.729 $\times \;10^{-9}\;T^{3}$ + 25,233 $T^{-1}$ − 7.6143 $\times 10^{-11}\;T^{4}$
$L_{Ti,Nb}$ (J/mol)	13,045.3
$L_{Ti,Zr}$ (J/mol)	−9321 + 11.9 T
$L_{Zr,Nb}$ (J/mol)	15,911 + 3.35 T
$L_{Ti,Zr,Nb}^{0}$ (J/mol)	−1000
$L_{Ti,Zr,Nb}^{1}$ (J/mol)	−13,000
$L_{Ti,Zr,Nb}^{2}$ (J/mol)	16,800
$V_{m}$ (m^3^/mol)	1.2496 ${\times 10}^{-5}$
$Q_{Ti,Nb}$ (J/mol)	−171,237.75–115.83 T
$Q_{Ti,Zr}$ (J/mol)	−131,670.56–133.36 T
$Q_{Zr,Nb}$ (J/mol)	−135,119.44–148.59 T
$Q_{Nb,Zr}$ (J/mol)	−358,612.72–84.43 T
$Q_{Zr,Zr}$ (J/mol)	−104,624.81–163.15 T
$Q_{Nb,Nb}$ (J/mol)	−395,598.95–82.03 T
$Q_{Ti,Zr,Nb}$ (J/mol)	14,510.81
$Q_{Ti,Nb,Nb}$ (J/mol)	113,467.29–32.01 T
$Q_{Zr,Nb,Nb}$ (J/mol)	43,010.37 + 74.52 T
$Q_{Ti,Zr,Zr}$ (J/mol)	41,384.1106–12.57 T
$Q_{Ti,Nb,Zr}$ (J/mol)	12,851.76
$Q_{Zr,Nb,Zr}$ (J/mol)	83,103.91 + 50.78 T
$a_{Ti}$ $a_{Zr}$ $a_{Nb}$ (nm)	0.3315 0.357 0.331

## Data Availability

Not applicable.
